# COVID-19 pandemic stressors and their longer-term association with young people’s wellbeing

**DOI:** 10.1371/journal.pone.0347875

**Published:** 2026-05-13

**Authors:** James Laurence, Emer Smyth

**Affiliations:** 1 UCL Social Research Institute, University College London, London, United Kingdom; 2 The Economic and Social Research Institute, Dublin, Ireland; Panteion University: Panteion Panepistemio Koinonikon kai Politikon Epistemon, GREECE

## Abstract

There is a significant body of research on adolescent wellbeing during the pandemic but less attention to the pathways through which the pandemic might be driving longer-term impacts on wellbeing. This paper addresses this gap using prospective cohort data collected at three time-points (ages 9, 12, 13; n = 2404; 50% female; 12.3% migrant-background) in Ireland, a country with a protracted period of school closures. Data collected from mid-2021 to mid-2022 (the late- to post-pandemic period) are used to analyse whether disruption across key domains such as education, family and peer relations during the pandemic has longer-term associations with adolescent self-reported mental wellbeing, while adjusting for socioemotional wellbeing before and during the pandemic. Several stressors (e.g., health-anxiety, support for home learning), which predicted worse wellbeing during the pandemic, were not significantly associated with late-/post-pandemic wellbeing. However, adolescents who, at the height of the pandemic, experienced greater disruption to familial relations and peer social relations (especially among girls), household economic shocks, elevated screen-time use, and a lack of a quiet place to study, report worse longer-term wellbeing outcomes. Some of these longer-term associations emerge from their link with worse wellbeing reported during the pandemic, which, in turn, predicts poorer longer-term wellbeing. However, screen-time and economic shocks remain linked with poorer late-/post-pandemic wellbeing even after accounting for peri-pandemic wellbeing. The findings highlight how external shocks such as the pandemic appear to be experienced differently depending on the social and economic resources of young people and their families, and how these can potentially shape adolescents’ wellbeing after the initial stressors have abated.

## Introduction

Extensive research documents how the pandemic harmed mental wellbeing, especially around its initial onset [1,2]. These negative outcomes emerged from multiple stressor-pathways, such as health anxiety, financial insecurity, loneliness, increases in childcare responsibilities, and family strains [3,4]. Although the pandemic has been challenging for all people, there is particular concern regarding its impact on young people’s mental wellbeing and longer-term life chances, given the social, psychological and educational disruption experienced during a key developmental stage [5,6]. Indeed, youth mental wellbeing worsened significantly during the pandemic, linked to stressors common across all ages, such as social disconnection, but also stressors unique to young people, such as school closures [5,7,8].

While research has documented how different types of stressors generated by the pandemic (hereafter, pandemic-stressors) were associated with young people’s mental wellbeing at the height of the pandemic, less research has explored the potential longer-term impacts of these stressors on their wellbeing. Some studies suggest young people’s wellbeing has remained persistently worse since the pandemic ended [9]. However, little work has examined which pandemic-stressor pathways may be driving any such longer-term negative impacts. Understanding which stressors experienced at the height of the pandemic remain linked with youth mental wellbeing into the late- and post-pandemic period is critical, especially given the importance of wellbeing in youth for shaping future life chances. Indeed, adolescence is increasingly recognised as a critical period for shaping long-term health and wellbeing trajectories, with global public health initiatives highlighting the importance of investing in adolescent health promotion and prevention strategies [10,11].

Using Ireland as a case study, this paper examines whether stressors experienced during the height of the COVID-19 pandemic have longer-term associations with young people’s mental wellbeing as they were transitioning out of the pandemic. Ireland was a country with a protracted period of school closures as well as prolonged prohibitions on social mixing. Prior work has demonstrated that, at the height of the pandemic, children’s wellbeing, especially among girls, was significantly affected by disruption across different life domains, such as disruption to education or curtailed contact with friends [12,13]. This paper explores whether these pandemic-stressors have continued to shape longer-term patterns of youth mental wellbeing. To investigate this question, we draw on longitudinal cohort data (Growing Up in Ireland) on young people born in 2008. We use three waves of data: when young people were surveyed at age nine (pre-pandemic); at age twelve, during the pandemic (December 2020); and at age thirteen, in 2021/22 (late- to post-pandemic period).

The central research questions are:

- Do experiences of pandemic-related stressors across different life domains at the height of the pandemic have any longer-term association with youth mental wellbeing during the late- to post-pandemic period, and do these associations remain after adjusting for indicators of pre-pandemic socio-emotional wellbeing?
- Do some stressors have more consistent links with longer-term wellbeing than others?
- And are there any gender differences in the associations between pandemic-stressors and later-/post-pandemic mental wellbeing?

This paper contributes to the literature on the impact of external shocks (such as pandemics or recessions) on adolescent wellbeing in three main ways. First, it shifts attention to the late/post-pandemic period in a country that experienced prolonged school closures (Irish schools spent 22 weeks fully closed compared to 16 weeks in UK, 14 in Germany, and 7 in France) and other public health restrictions, investigating whether there is evidence of potential scarring effects of exposure to protracted stress. Second, it exploits variation in experience of pandemic stressors, driven by differences in economic and social resources, to look at the associations between different stressors and longer-term poorer wellbeing. Third, it focuses on a key development phase during which young people experienced other disruptions including the transition to secondary education, potentially making them more vulnerable to the additional stressors of the pandemic. Given wellbeing in adolescence can shape longer-term life outcomes and adaptive development [14], understanding whether (and which) pandemic-stressors may still be linked with their wellbeing is crucial to ameliorating any persistent harm. In the following section we place this study in the context of previous literature on the pandemic and adolescent wellbeing.

## Literature review

### The pandemic and youth mental wellbeing

Worsening mental wellbeing during the pandemic appears evident to some degree among all social groups [1]. However, young people may have experienced particularly acute impacts, especially given the link between experiences of interpersonal stress and the emergence of emotional difficulties in youth [5]. Youth, and adolescence in particular, involves key developmental changes, which can increase the risk of social-emotional disorders, especially in response to stressors, which persist over the life course [5,15,16]. Accordingly, the pandemic may have had a lasting impact on youth wellbeing. However, at the same time, set-point theories of mental wellbeing, alongside resilience frameworks in developmental psychology, emphasise that individuals may adapt and recover following adversity, meaning short-term declines in wellbeing may diminish over time as stressors abate [17,18]. The following literature review will consider whether pandemic-driven stressors are likely to leave a scar on young people’s mental wellbeing by drawing on theories and empirical findings relevant to the relationship between youth developmental stage, stress and wellbeing, but embedding this research in studies on young people’s pandemic experiences.

Theoretically, there are several reasons young people’s mental wellbeing may have been more susceptible to the pandemic. Disconnection from peers may have been particularly harmful for young people given their greater reliance on peer connections for social development and emotional support [5,6]. Young people also exhibit greater concerns about maintaining close connections to friends, and isolation during the pandemic may have had keener consequences for their social status, peer belonging, and subsequent mental wellbeing [6]. Youth is also a key developmental period during which decision-making and emotional regulation skills are developed, potentially rendering them more susceptible to stressors, coupled with a weaker ability to emotionally self-regulate [19]. Parents also play a critical role in young people’s adjustment and mental wellbeing; for example, through parenting styles and quality of parent-child relationships. Prolonged periods at home may have had especially negative impacts on youth wellbeing where parenting styles were more conflictual or quality of parent-child relationships poorer [6]. Youth is therefore a key developmental stage where issues with mental wellbeing can increase, potentially increasing susceptibility to the negative effects of the pandemic.

Studies demonstrate how the mental wellbeing of children and young people, ranging from 4- to 18-years-old, worsened significantly during the pandemic. This includes increases in emotional/behavioural difficulties [7,8,20–22], higher depressive symptoms and anxiety, and worse life satisfaction [5,7]. In addition, some groups of young people may have experienced worse outcomes, including children from poorer households, those with special educational needs, or those with neurological conditions [5,20,21,23]. As in the general population, female youth experienced particularly acute mental wellbeing changes [5,7,20,23]. Studies suggest this could stem from girls being more likely to internalise problems [24] or rely more on peers for emotional support [25]. Meta-analyses document stronger effects among adolescents [20].

These negative changes in mental wellbeing over the pandemic are linked to a range of stressors which were triggered by the pandemic [3]. One of the most consistent pandemic-stressor pathways is reduced social connectivity, where separation from peers, lack of face-to-face interaction, and feelings of loneliness significantly worsened mental wellbeing [5–8,22]. Indeed, not being able to take part in normal extra-curricular activities or attend social events were the highest COVID-related anxieties reported by young people [5]. However, other pandemic-stressors, such as anxiety about the health of family and friends [5], and worries about the financial situation of the family [6], were also linked to worse mental wellbeing, while increasing screen time among young people during the pandemic is also linked to worsening wellbeing [26].

In addition to these more general pandemic-stressors, young people experienced a separate, relatively unique set of stressors. Parent-child relationships emerged as a key pathway of poorer wellbeing, given school closures meant large periods of time were spent at home. Those who experienced more conflict with their parents saw larger declines in their mental wellbeing [5], while those who spent more time doing activities with family, or felt supported by family, saw less harm [7,21]. Higher parental stress and worse partner relationships were linked to worsening wellbeing among young people [21,22]. Young people also experienced stressors linked to education. Some adolescents, especially those from disadvantaged backgrounds, found transitioning to remote learning stressful, while others experienced difficulties in online learning, harming mental wellbeing [5]. Similarly, worries about time lost on schooling was linked to more depression [6].

These questions on youth wellbeing during the pandemic, and potential processes of scarring, resonate with broader resilience frameworks in the psychology literature. Resilience research emphasises how individuals respond to adversity through the interaction of risk factors (e.g., economic shocks, family conflict, social isolation) and protective resources such as supportive relationships, stable environments, and opportunities for social engagement [27–29]. From this perspective, large-scale disruptions such as the COVID-19 pandemic represent a significant external stressor, but their longer-term impact on young people’s wellbeing is likely to vary depending on the arrangement of risk and protective factors in their social environments. These factors may determine both *if* an individual experiences a certain stressor, and if they do, *how deeply* the stressor impacts their mental wellbeing. While some stressors may produce persistent negative effects, other young people may demonstrate resilience through adaptation and recovery once restrictions and disruptions ease [5,8,30].

### The long-term impact of the pandemic on mental wellbeing

Research thus documents what stressors shaped youth mental wellbeing at the height of the pandemic. However, less is known about whether these stressors had any longer-term impacts. Potentially, the pandemic might have had little long-term impact on youth wellbeing. Many pandemic-stressors significantly weakened from mid- to late-2021 onwards. By mid- to late-2021, mixing restrictions in most societies had been fully lifted. Most schools (and universities) were fully reopened in 2021, allowing young people back into education. This, in turn, reduced the pressures of childcare, homeschooling and managing work-life balance for families. Transitioning back to school may also have reduced young people’s exposure to more difficult family relations. As the economy recovered, unemployment declined, and financial insecurity linked to the pandemic also declined (although the cost-of-living crisis likely had a growing impact). Accordingly, as pandemic-linked stressor pathways abated, their impact on mental wellbeing may have also weakened, resulting in little long-term impacts. Adaptation to the ‘new normal’ of the pandemic, with compensatory means of communication or a sense of ‘togetherness’ acting as sources of resilience, may have also prevented longer-term harm [18]. In addition, set-point theories of wellbeing suggest that, even during times of crisis, shorter-term fluctuations in mental wellbeing can quickly return to their prior level [17].

Yet, experiences during the pandemic may also have left a lasting scar on youth wellbeing. Having had a close friend or family member hospitalised or die is linked to higher depression [31], which could lead to longer-term mental ill health. Despite the lifting of social restrictions, ‘vulnerability–stress’ models suggest repeated experiences of stress can have a compounding, long-term negative impact on mental wellbeing [16]. Accordingly, repeated lockdowns and attendant social isolation could lead to “a worsening of mental wellbeing in the long run, especially in vulnerable groups” [[32], p.290]. Studies of previous pandemics show negative long-term effects of quarantine, which can remain after isolation ended [33]. Furthermore, as outlined, child-parent conflict during lockdowns was a key stressor, and greater time spent in such environments may scar young people’s mental wellbeing [34]. The pandemic may have also affected young people’s opportunities for growth and achievement via reduced school attendance or impaired school performance, continuing to depress mental wellbeing even after school returns [35]. There is some evidence screen time use has remained elevated since the onset of the pandemic among young people [36], which can lead to long-term internalising symptoms [37]. Long-term impacts of unemployment and income loss can also harm families’ mental wellbeing, in turn, scarring young people’s wellbeing [38].

Overall, there is extensive evidence that periods of mental ill health do not necessarily diminish after the initial stressor is removed. Instead, processes can sustain a continuity in ill health, “including relatively persistent changes in beliefs about self and the world and alterations in patterns of behaviour that have knock-on experiential consequences, such as avoidance of experiences that might disconfirm threat beliefs or stimulate the development of coping skills” [[33], p.1376].

Available longer-term evidence of youth mental health since the pandemic shows that mental wellbeing has not fully recovered. Montero-Marin et al. [7] found poorer mental wellbeing still evident among 13-year-olds when returning to school after closures. A meta-analysis of children/adolescents found the prevalence of mental ill health increased and then stabilised over the pandemic up to at least January 2022 [9]. In the Netherlands, Zijlmans et al. [39] tracked children (ages 8–18) from April 2020 to April 2022 and found internalising problems increased over the period, and were higher by April 2022 than at the start of the pandemic. An analysis of German youth (7–17) found that post-pandemic (September-October 2022) mental wellbeing had recovered somewhat since the pandemic, although it remained worse than before the pandemic. Henseke et al. [40] found that between February 2021 to May 2022, British young people’s (aged 16–25) life satisfaction significantly improved. In contrast, the life satisfaction of similarly aged German adolescents continued to remain depressed two years after the pandemic began [41]. In sum, current evidence suggests there has potentially been long-term harm to youth wellbeing (although as the pandemic was ending many countries were experiencing additional crises (e.g., war in Ukraine, cost-of-living crisis), which may also shape youth mental wellbeing – discussed further below).

Prior longitudinal work in Ireland (the case study examined here), using pre- and peri-pandemic measures of mental wellbeing, shows how young adults (20-years-old) [12] and mothers [4] saw mental wellbeing decline during the pandemic. Analyses of the experiences of children (aged 12) in Ireland found a range of pandemic-driven changes in their lives were associated with poorer mental wellbeing; in particular, where children increased their amount of screen-time, argued more with their parents, had less contact with their friends, were anxious about the virus, and were living in a household that experienced a drop in income [12,13]. In addition, children who lacked a quiet place to study or a computer on which to engage in remote learning also reported poorer wellbeing [13]. From a resilience perspective, such mixed patterns of recovery may reflect variation in young people’s exposure to pandemic-related stressors as well as differences in the social and economic resources available to cope with them.

## Overview of the study

Taken together, studies demonstrate that youth mental wellbeing significantly worsened during the pandemic, and that wellbeing appears to have remained depressed in the late- to post-pandemic period, raising important questions about resilience and longer-term recovery from pandemic-related stressors. However, less research has looked at whether, and which, pandemic-stressors may have longer-term associations with youth mental wellbeing. This study aims to examine whether young people’s stressor-experiences at the height of the pandemic across different domains of their lives exhibit any lasting associations with their mental wellbeing in Ireland.

The theories and evidence reviewed above lead to competing predictions of whether stressors experienced during the pandemic will exhibit longer-term associations with youth wellbeing or not. Therefore, we develop a series of research questions (not hypotheses) that will be explored in this study. The first research question therefore is: do stressors experienced at the height of the pandemic (peri-pandemic stressors) have any longer-term association with youth mental wellbeing as the country was transitioning out of the pandemic (late-/post-pandemic period)? The current literature also suggests certain pandemic-stressors (e.g., social disconnection) may be more acutely related to wellbeing than others (e.g., educational disruption). The second research question therefore is: which pandemic-stressors are more likely to be associated with longer-term youth mental wellbeing? The literature reviewed also suggests girls and boys may have experienced different stressors, and certain stressors more acutely, leading to gender differences in how the pandemic is associated with youth mental wellbeing in the longer-term. The third research question therefore is: are there gender differences in the longer-term associations between pandemic-stressors and mental wellbeing? In the following section we outline the data, measures and methodological approach.

## Data and methods

### Data

This study uses data from the 2008 Cohort of the Growing Up in Ireland (GUI) study (50% female; 12.3% migrant background). This prospective cohort study follows a representative sample of children born in 2008 (between 1st December 2007 and 30th June 2008), alongside their primary and secondary caregivers. Households were first surveyed when the children were nine-months-old, sampled on the basis of the child benefit register. This study uses three waves of GUI data. The first wave used is a pre-pandemic wave (June 2017-February 2018), gathering data in face-to-face interviews on children and households when they were nine years old (72 per cent response rate of initial sample). The second wave constitutes the pandemic-period wave (45 per cent response rate, which is similar/superior to well-established longitudinal surveys conducted over the pandemic, e.g., UK Household Longitudinal Study – 48 per cent – Millennium Cohort Study – 29 per cent). This was conducted in December 2020, when children were around twelve, collecting data on the young people, and their mothers, using an online survey relating to their experiences of the pandemic (the switch to online modes being the norm among all pandemic-period surveys). The cohort was subsequently interviewed by telephone around a year or so later (July 2021-June 2022), at 13 years of age, when they were adolescents. The response rate was 78 per cent of the valid sample (that is, of those who took part at the age of nine) and 57 per cent of those who took part when the child was 9-months-old [42]. This is taken to constitute the ‘late- to post-pandemic’ wave, capturing young people’s experiences as the country was transitioning out of the pandemic. While there were some restrictions on organised events in Ireland for the early part of the fieldwork period, schools had re-opened, most day-to-day activities had resumed and vaccines had been rolled out for teenagers. Despite a COVID-19 spike in cases in January 2022, schools re-opened as normal after the Christmas period. For young people at least, the survey was conducted at a time of minimal education restrictions, and when the country itself was completing its transition into the post-pandemic period. Listwise deletion of missing data was applied. Longitudinal weights are applied to address attrition and correct sample representativeness. Given wellbeing is our central focus, we considered that using multiple imputation might distort the results. The loss of cases due to missing values on the explanatory variables was also small (56 for MHI-5 and 35 for SMFQ) so multiple imputation was not justified. The data were first accessed for analysis 22-03-2024. The authors had no access to information that could identify individual participants during or after data collection.

While the GUI study gathers information from mothers and fathers, the young person is seen as the primary focus of the research with a strong emphasis on involving them in consultations about what is included in the study and on capturing their own perspectives on their lives [42]. Regarding children and young people as active agents in their own lives is now increasingly recognised in research in general [44] and in cohort studies especially [45]. The analyses presented here therefore focus on outcomes and pandemic experiences reported by young people themselves, though information collected from mothers is used to capture socio-demographic factors and socio-emotional wellbeing of children when they were too young to provide such information themselves (see Table 1).

**Table 1:** Descriptive statistics.

	%	Mean		
		(Std. dev.)	Reported by:	Mode
*Outcome variables (age 13)*				
Mental Health Inventory 5		77.4 (15.9)	YP	Phone
Short Moods and Feelings Questionnaire		6.0 (5.8)	YP	Online
*Explanatory variables (age 9)*				
Female	49.6		M	Face-to-face
Mother’s education:			M	Face-to-face
Lower secondary or less	9.2			
Upper secondary (Leaving Certificate)	14.2			
Post-secondary	43.4			
Degree or higher	33.2			
Income at age 9:			M	Face-to-face
Quintile 1	15.3			
Quintile 2	18.2			
Quintile 3	18.5			
Quintile 4	19.6			
Quintile 5	18.1			
Income missing	10.3			
Urban	43.6		M	Face-to-face
Migrant background	12.3		M	Face-to-face
Lone-parent family	15.0		M	Face-to-face
YP hampered by disability at 9	12.3		M	Face-to-face
2^nd^ year in second-level school	65.2		YP	Phone
Neighbourhood disorder at 9		6.8 (2.5)	M	Face-to-face
Family living locally	59.8		M	Face-to-face
SDQ externalising behaviour at 9		4.2 (3.3)	M	Face-to-face
SDQ internalising behaviour at 9		3.2 (3.1)	M	Face-to-face
*Pandemic experiences*				
Quiet place to study	50.1		YP	Online
Suitable device/computer	74.7		YP	Online
Support for learning at home	61.0		YP	Online
Fall in household income	35.1		M	Online
Received PUP	32.2		M	Online
Parents working remotely:			M	Online
One	29.7			
Both	9.0			
Sees friends (Ref. About the same):			YP	Online
More often	17.5			
Less often	44.6			
Uses screen for fun more than previously	60.3		YP	Online
Engaged in sports:			YP	Online
More often	20.3			
Less often	34.6			
Argue with parents more than usual	43.3		YP	Online
Argue with siblings more than usual	50.9		YP	Online
Worried about family being infected:			YP	Online
Always true	36.3			
Sometimes true	48.8			
MHI5 during pandemic		73.7 (16.2)	YP	Online

Notes: Phone – telephone interview; online – online self-completion; face-to-face – in-person interview; YP – young person, M – mother (primary caregiver).

### Outcomes

Table 1 provides details on the outcome/explanatory variables used in the analyses as well as indicating the source of the information (the young person or their mother) and the mode of data collection (face-to-face, telephone or online self-completion). The measures of wellbeing gathered in the data changed between waves of the study to reflect the developmental stage of the young person, an approach that is adopted by cohort studies internationally.

To measure late-/post-pandemic mental wellbeing, two outcome scales at age 13 are considered: the Mental Health Inventory 5 (MHI-5) measure of overall wellbeing (e.g., ‘have you been a very nervous person’) and the Short Moods and Feelings Questionnaire (SMFQ), designed to capture depressive symptoms (e.g., ‘I felt miserable or unhappy’). These measures have a reliability of 0.78 and 0.92 respectively [[42]; see also for full items in the scales]. The measures differ in that the MHI-5 includes some items on more positive affect while the SMFQ focuses on negative feelings. There is also a mode difference, with the MHI-5 collected through a telephone interview and the SMFQ through online self-completion. The fact that the MHI-5 was collected over the phone may affect the responses if a young person is reluctant to acknowledge negative feelings to an interviewer so this must be regarded as an upper-bound estimate.

To measure pre-pandemic mental wellbeing (age 9), measures of internalising and externalising behaviour (based on the Strengths and Difficulties Questionnaire, SDQ) were used, as the MHI-5 and SMFQ scales had not been administered at this wave (given they are not designed to capture wellbeing at 9-years-old). As outlined, the focus of this study is not to track changes in wellbeing over the pandemic. Instead, it seeks to examine whether pandemic-stressors remain associated with wellbeing in the late-/post-pandemic period. In addition, measuring young people’s self-reported wellbeing has the advantage of not relying on parental views of what their children are experiencing. Accounting for pre-pandemic SDQ will go some way to ensuring that poorer wellbeing in the wake of the pandemic is not solely related to pre-existing socio-emotional difficulties. Such an approach has been widely used with other international cohort study data (e.g., Terhaag et al., 2021). The MHI-5 measure was administered via an online survey in the pandemic-period wave (age 12, December 2020), providing a measure of wellbeing in the wake of the first set of public health restrictions. These measures have been used internationally and show strong validity. Previous studies have shown configural, metric, scalar, and residual invariance in the overall SDQ measure and its subscales for a large UK sample from ages 5–14, and in the SMFQ measure for a large UK sample from adolescence into adulthood. Similarly, the MHI-5 has shown good consistency and construct validity with adolescent samples [43,46,47].

### Pandemic stressors

This study examines a range of stressors that young people experienced during the pandemic. This includes measures relating to remote education, family economic circumstances, contact with friends, day-to-day activities, and family relationships.

Educational experiences during the first period of school closures are captured by three measures: whether it was ‘always true’ (as opposed to ‘sometimes true’ or ‘never true’) that the young person had a quiet place to study at home, whether they had access to a computer or device needed for study and whether someone at home helped them with their schoolwork. A significant proportion of young people did not have support for online learning during the first lockdown: a quarter did not have a suitable device for home learning, half did not have a quiet place to study and four-in-ten did not have someone at home to help with schoolwork (Table 1). These patterns varied by social background and migrant background [13,52].

For many families, the pandemic represented an economic shock with the closure of workplaces. A Pandemic Unemployment Payment (PUP), paid at a higher rate than normal unemployment payments, was put in place to provide a financial cushion. Over a third (35%) of households reported a pandemic-related fall in income while just under a third received PUP (Table 1). In 38 per cent of cases, one or both parents shifted to working remotely during the first lockdown.

Reduced contact with friends during the pandemic is measured by whether young people reported seeing their friends more, less or about the same as before the pandemic. Even though schools had re-opened by December 2020, 45 per cent of young people were seeing their friends less often than before the pandemic. The pandemic restrictions are also linked with young people’s day-to-day engagement in other activities such as sports, that have been found to have a protective effect on wellbeing. The analyses thus examine whether young people took part in sport more or less than before the pandemic, compared to the same level of sports activities: 35 per cent had reduced their involvement but 20 per cent were taking part in sports more often than before. A measure of increased screen time compared to the pre-pandemic era is also considered, with the majority (60%) increasing their use of screens for fun.

Family dynamics are captured by using young people’s reports of whether they argued with their parents or siblings more than usual and whether they had been worried about their family being infected by the COVID-19 virus. A significant proportion argued more with family members than previously – 43 per cent with parents and 51 per cent with siblings. Furthermore, the vast majority of young people worried, at least sometimes, about family being infected with COVID-19.

### Covariates

Measures of individual/family background were captured at the pre-pandemic period, including gender, maternal education, equivalised household income (divided into quintiles), whether the family had one or two resident parents, whether the parents were from outside Ireland (migrant background), whether the family lived in an urban area, potentially linked with access to nature [48], and whether the young person had a long-standing illness or disability which hampered their day-to-day life. These covariates are selected given prior research demonstrating their importance for young people’s mental wellbeing in Ireland [49]. In addition, these covariates may also be associated with one’s likelihood of experiencing pandemic-stressors (e.g., young people in lower socio-economic status households may experience more household economic stressors or lack a quiet place to study or device to study on) [e.g., [5]]. Robustness checks were conducted to assess whether the patterns changed when income and family structure at age 13 (late-/post-pandemic period) are considered. To capture resources and challenges in the local neighbourhood, whether the household has family living locally is included as is a composite measure of neighbourhood disorder (based on the frequency of problems like vandalism, public drinking, and drug-taking). A dummy variable is included to capture whether the young person is in first or second year of secondary education.

### Modelling and analytical approach

To investigate whether pandemic-period stressors are associated with late-/post-pandemic wellbeing, the analyses use nested OLS regression models for both late-/post-pandemic depressive symptoms (SMFQ) and positive wellbeing (MHI-5) separately. Model 1 examines the association between indicators of children’s social, economic, and demographic status at age 9 (pre-pandemic) and their mental wellbeing outcomes at age 13 (late-/post-pandemic) to see how patterns of mental wellbeing differ across groups in the late-/post-pandemic period (as noted, robustness checks were conducted to assess whether the findings changed when income and family structure at age 13 are taken into account but results using age 9 indicators remain present). Model 2 then includes two measures of pre-pandemic wellbeing at age 9: internalising and externalising socio-emotional difficulties, to examine whether any late-/post-pandemic gaps across groups persist after adjusting for pre-pandemic wellbeing. Model 3 then tests whether stressors experienced during the pandemic continue to predict young people’s late-/post-pandemic mental wellbeing outcomes one year later, after adjusting for pre-pandemic mental socio-emotional wellbeing. Lastly, there are two ways experiences of pandemic-stressors may have a long-term relationship with adolescent mental wellbeing. The first is that stressors could be linked with poorer mental wellbeing *during* the pandemic itself, and worse mental wellbeing outcomes during the pandemic may lead to poorer longer-term mental wellbeing outcomes. The second way is that even after accounting for the link between stressors and mental wellbeing during the pandemic, stressor-experiences may be linked with long-term scarring of mental wellbeing, continuing to affect mental wellbeing into the late- to post-pandemic period. To test these scenarios, Model 4 includes a measure of wellbeing (MHI-5) during the pandemic.

**Fig 1:**
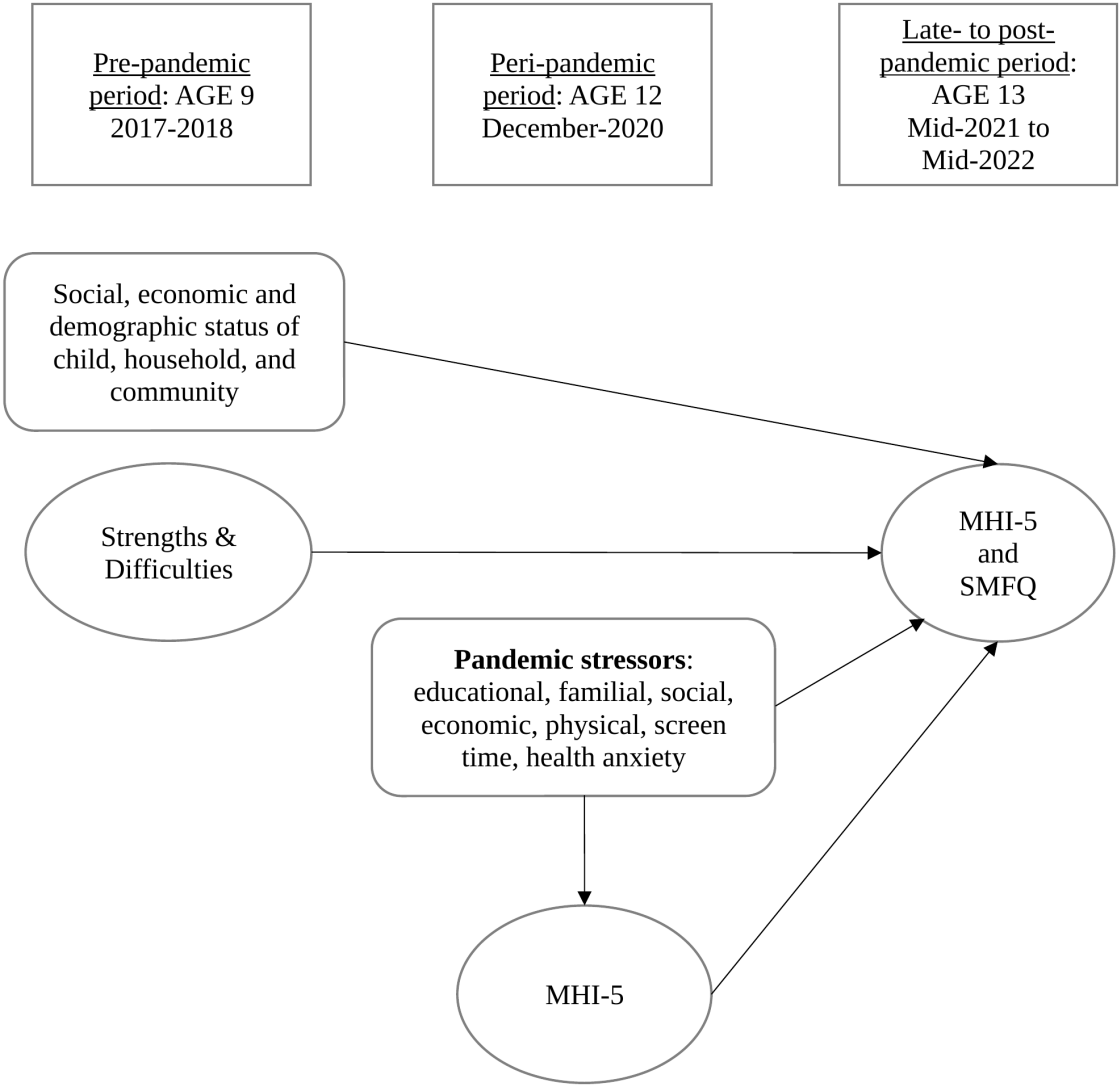
Outline of key variables and time-periods measured.

As outlined, current evidence suggests girls experienced worse pandemic mental wellbeing outcomes than boys. The final analytic stage will examine whether pandemic-stressors may have different long-term associations among girls and boys. While the data structure prevents the application of more causally robust modelling (e.g., fixed-effects), the OLS approach allows us to test whether stressors experienced during the pandemic are associated with mental wellbeing in the late-/post-pandemic period, after adjusting for individual and family factors as well as wellbeing before and during the pandemic. Fig 1 summarises the study approach, demonstrating the key groups of variables analysed, the time-points at which they are measured, and highlights the key relationships that are examined (not the full potential interrelationships between all variables applied in the study).

## Results

### The long-term associations between pandemic-stressors and late-/post-pandemic mental wellbeing

We begin by examining whether stressors experienced at the height of the pandemic exhibit longer-term associations with youth mental wellbeing, and if so, whether there is heterogeneity in associations across different types of stressor (research questions 1 and 2). The first analyses explore late-/post-pandemic wellbeing (MHI-5) among adolescents, where higher values indicate more positive mental wellbeing outcomes. Model 1 (Table 2) demonstrates substantial gender differences, with much lower wellbeing among girls: a difference of over half a standard deviation. There is little significant variation by parents’ education or household income, but lower wellbeing is found among adolescents in lone-parent families, among those living in urban areas, and among adolescents with a disability. There is no systematic variation by neighbourhood disorder or by whether family members live in the locality.

**Table 2:** Factors associated with late-/post-pandemic wellbeing (MHI-5) at age 13.

	Model 1	Model 2	Model 3	Model 4
*Late-/post-pandemic Outcome:*	MHI-5	MHI-5	MHI-5	MHI-5
Female	−8.187***	−8.474***	−8.202***	−5.878***
Mother’s education:				
Leaving Certificate	0.349	−0.063	0.361	0.494
Post-secondary	−0.997	−0.915	−0.296	0.212
Degree or higher	−2.102	−2.183	−0.935	−0.299
Income at age 9:				
Quintile 2	0.274	0.349	−0.078	0.017
Quintile 3	−0.083	−0.074	−0.122	0.247
Quintile 4	1.878	1.759	1.811	1.975
Quintile 5	1.062	0.894	1.162	1.675
Income missing	0.197	0.125	−0.170	−0.409
Urban	−3.840***	−3.739***	−3.713***	−2.795***
Migrant background	−1.836	−1.593	−1.818	−1.195
Lone-parent family	−5.406**	−4.214*	−4.453*	−3.376*
YP hampered by disability	−4.146**	−1.395	−1.782	−1.028
2^nd^ year in second-level school	−0.943	−0.783	0.024	1.251
Neighbourhood disorder at 9	0.272	0.455**	0.528**	0.451**
Family living locally	0.230	0.344	0.153	−0.463
SDQ externalising behaviour at 9		−0.152	−0.036	−0.027
SDQ internalising behaviour at 9		−0.886***	−0.848***	−0.555**
*Pandemic experiences*				
Quiet place to study			1.730	0.008
Suitable device/computer			1.756	0.036
Support for learning at home			0.238	−0.629
Fall in household income			−3.325**	−2.125*
Received PUP			2.204*	1.153
Parents working remotely:				
One			−0.886	0.034
Both			−1.816	−1.016
Sees friends (Ref. About the same):				
More often			0.216	0.428
Less often			−1.895*	−1.292
Uses screen for fun more than previously			−1.413	−0.334
Engaged in sports:				
More often			0.612	0.619
Less often			−0.404	−0.376
Argue with parents more than usual			−2.992**	−0.367
Argue with siblings more than usual			0.728	1.204
Worried about family being infected:				
Always true			−0.721	1.463
Sometimes true			−0.569	0.419
MHI5 during pandemic				0.407***
Constant	83.693***	85.351***	84.781***	50.034***
Observations	2404	2403	2403	2348
R-squared	0.112	0.139	0.172	0.297

Notes: OLS regression (robust standard errors)

*** p<.001, ** p<.01, * p<.05; YP=young person

Model 2 (Table 2) demonstrates that adolescents with more pre-pandemic socio-emotional difficulties (SDQ) have lower late-/post-pandemic wellbeing (although this association is only significant for internalising problems). Model 2 also shows that gender, living in an urban area, and being in a lone parent household before the pandemic continue to predict wellbeing even after adjusting for pre-pandemic socio-emotional difficulties, suggesting gaps in wellbeing between these groups may be larger in the late-/post-pandemic period. However, the lower wellbeing among adolescents with a disability is rendered non-significant after adjusting for pre-pandemic socio-emotional difficulties. The coefficient for neighbourhood disorder, meanwhile, becomes significant and positive when including prior socio-emotional difficulties. Further analyses indicate that this finding relates to higher prior internalising and externalising behaviour in more disorderly neighbourhoods. Potentially, this group may have seen smaller changes in their wellbeing post-pandemic because they already had more pre-pandemic difficulties.

Model 3 (Table 2) tests whether stressors experienced during the pandemic have a longer-term association with adolescent wellbeing in the late-/post-pandemic period. Educational experiences during the first period of school closures, such as having a quiet place to study or support for learning at home, are not significantly related to later wellbeing. However, economic experiences appear important. Adolescents in households that saw a fall in household income during the pandemic have significantly worse wellbeing in the late-/post-pandemic period, while being in a household which received a Pandemic Unemployment Payment (PUP) is associated with higher wellbeing.

Pandemic stressors related to social relationships are also linked with long-term differences in wellbeing. Those who, during the pandemic, reported seeing friends less often than before the pandemic (even though schools had reopened by December 2020), and those who reported arguing more than usual with their parents, continued to report lower late-/post-pandemic wellbeing. Meanwhile, adolescents who, during the pandemic, argued with siblings more than usual, were worried about their family being infected, engaged in sports less often, and who used screens more than previously, do not significantly differ in their later wellbeing.

Model 4 explores whether any of the observed long-term associations between pandemic-stressors and late-/post-pandemic wellbeing might be explained by the link between pandemic-stressors and wellbeing *during* the pandemic. Separate analysis shows that adolescents who reported negative peer and parental relations during the pandemic reported lower wellbeing during the pandemic itself, after adjusting for pre-pandemic socio-emotional difficulties (see S1 Appendix). Model 4 (Table 2) includes a measure of adolescent wellbeing during the pandemic. On doing so, the associations between family or peer stressors and late-/post-pandemic wellbeing are rendered non-significant. In other words, being isolated from friends and arguing with parents appears linked to lower late-/post-pandemic wellbeing because they are linked to reduced wellbeing during the pandemic, which, in turn, is linked to lower wellbeing a year or so later. In contrast, a drop in household income is found to have a negative association with wellbeing both during the pandemic (S1 Appendix) and in the late-/post-pandemic period, only partially mediated via pandemic-period wellbeing.

The second set of analyses explore late-/post-pandemic depressive symptoms (SMFQ) among adolescents, where higher values indicate more negative mental wellbeing (Table 3). There are common patterns but also important differences in the factors linked to depressive symptoms compared to wellbeing. There are again large gender differences, with girls exhibiting more depressive symptoms, by over half a standard deviation (Table 3, Model 1). As with wellbeing (MHI-5), there is little systematic variation by pre-pandemic measures of family background. However, living in an urban area and being in second year of secondary school are linked to more depressive symptoms while having family living locally is associated with fewer. Again, pre-pandemic socio-emotional difficulties are strongly linked to late-/post-pandemic depressive symptoms, but here the association is driven by externalising, rather than internalising, behaviour (Model 2).

**Table 3:** Factors associated with late-/post-pandemic depressive symptoms (SMFQ) at age 13.

	Model 1	Model 2	Model 3	Model 4
*Late-/post-pandemic Outcome:*	SMFQ	SMFQ	SMFQ	SMFQ
Female	3.672***	3.826***	3.798***	3.023***
Mother’s education				
Leaving Certificate	−0.348	−0.186	−0.203	0.014
Post-secondary	−0.036	0.019	0.224	0.369
Degree or higher	0.459	0.600	0.666	0.579
Income at age 9:				
Quintile 2	−0.586	−0.502	−0.398	−0.525
Quintile 3	−0.310	−0.253	−0.181	−0.377
Quintile 4	−0.115	−0.029	−0.015	−0.204
Quintile 5	−0.180	−0.059	−0.137	−0.267
Income missing	−0.378	−0.387	−0.344	−0.066
Urban	0.818*	0.767*	0.727	0.575
Migrant background	0.097	−0.023	−0.233	−0.351
Lone-parent family	1.046	0.768	0.952	0.684
YP hampered by disability	1.452	0.590	0.585	0.494
2^nd^ year in second-level school	0.871*	0.818*	0.660	0.256
Neighbourhood disorder at 9	0.070	−0.000	−0.022	0.013
Family living locally	−1.022*	−1.053**	−1.029**	−0.742*
SDQ externalising behaviour at 9		0.141*	0.066	0.050
SDQ internalising behaviour at 9		0.166	0.124	0.037
*Pandemic experiences*				
Quiet place to study			−1.289***	−0.534
Suitable device/computer			−0.687	−0.360
Support for learning at home			−0.402	0.131
Fall in household income			0.306	−0.041
Received PUP			−0.582	−0.227
Parents working remotely:				
One			0.054	−0.304
Both			0.003	−0.175
Sees friends (Ref. About the same):				
More often			0.447	0.498
Less often			0.034	−0.328
Uses screen for fun more than previously			1.435***	0.973**
Engaged in sports:				
More often			0.518	0.420
Less often			0.101	0.105
Argue with parents more than usual			1.066**	−0.012
Argue with siblings more than usual			0.345	0.373
Worried about family being infected:				
Always true			−0.046	−0.898
Sometimes true			−0.859	−0.998
MHI5 during pandemic				−0.152***
Constant	3.349**	2.695*	3.416**	15.943***
Observations	1639	1639	1639	1604
R-squared	0.141	0.157	0.220	0.350

Notes: OLS regression (robust standard errors)

*** p<.001, ** p<.01, * p<.05; YP=young person

Model 3 tests again whether peri-pandemic stressor-experiences have any long-term associations with depressive symptoms. While educational experiences during the first lockdown were not significantly related to later wellbeing, adolescents who had a quiet place to study during the pandemic report fewer depressive symptoms later on (Model 3). Unlike for wellbeing (MHI-5), family income shocks during the pandemic do not have a longer-term association with depression. Neither does seeing friends less during the pandemic. However, adolescents who spent more time on screens during the pandemic report higher depressive symptoms (while no association was observed for wellbeing). As with wellbeing, arguing with parents more than usual during the pandemic is associated with more depressive symptoms.

As above, one possibility is that the long-term associations between pandemic-stressors and depressive symptoms could be operating through their association with mental wellbeing during the pandemic itself. Indeed, greater screen time, arguing with parents, and not having a quiet place to study during the pandemic are all associated with worse mental wellbeing outcomes during the pandemic after adjusting for pre-pandemic socio-emotional difficulties (S1 Appendix). Model 4 therefore adds in adolescents’ peri-pandemic wellbeing, which renders having a quiet place to study and arguing with parents non-significant, suggesting their longer-term association with late-/post-pandemic depressive symptoms comes through their link with mental wellbeing during the pandemic itself (as noted, depressive symptoms (SMFQ) were not measured among young people during the pandemic; only wellbeing (MHI-5), thus Model 4 can only adjust for pandemic-period wellbeing as an indicator of mental health). However, increased screentime during the pandemic continues to have an association with later depressive symptoms, even after pandemic-period wellbeing is taken into account.

### Gender differences in the long-term associations between pandemic-stressors and late-/post-pandemic mental wellbeing

Thus far, the study has explored the longer-term associations between stressors experienced during the pandemic and mental wellbeing in the late-/post-pandemic period, identifying key heterogeneity across stressor-type (research questions 1 and 2). In addition, it has examined how far such longer-term associations with late-/post-pandemic mental wellbeing are linked with worse wellbeing experienced during the pandemic itself. However, as outlined in the review of the literature, there are reasons to think pandemic-stressors may be differently linked with male and female adolescent wellbeing (research question 3). In other words, the results for all adolescents outlined above may mask important heterogeneity in longer-term associations across genders.

The final analyses therefore examine gender differences in the drivers of late-/post-pandemic depression and wellbeing. To do so, we replicate Model 4 (Table 2) and Model 4 (Table 3), examining wellbeing and depressive symptoms respectively, but split the sample between boys and girls (Models 1–4, Table 4). Several key findings emerge. Firstly, wellbeing (MHI-5) during the pandemic has a stronger positive association with late-/post-pandemic wellbeing among girls than boys (Model 2 compared to Model 1, Table 4), and a stronger negative association with late-/post-pandemic depressive symptoms among girls than boys (Model 4 compared to Model 3, Table 4). Tests for whether these differences were statistically significant showed that pandemic-period wellbeing has a significantly stronger positive association with late-/post-pandemic wellbeing among girls (the difference was not significant for depressive symptoms).

**Table 4:** Full models (Model 4) for late-/post-pandemic wellbeing and depression at age 13, separately for males and females.

	Model 1	Model 2	Model 3	Model 4
	Males	Females	Males	Females
*Late-/post-pandemic Outcome:*	MHI-5		SMFQ	
Mother’s education:				
Leaving Certificate	2.451	−0.369	−0.191	0.927
Post-secondary	−0.482	1.216	0.364	1.107
Degree or higher	−1.502	1.377	0.339	1.533
Income at age 9:				
Quintile 2	0.849	−1.806	−0.559	−0.171
Quintile 3	3.689	−2.862	−0.499	−0.183
Quintile 4	2.021	1.191	0.160	−0.356
Quintile 5	1.547	1.473	0.517	−0.976
Income missing	−0.196	−1.139	−0.012	0.057
Urban	−1.090	−4.093**	0.261	0.740
Migrant background	−1.349	−1.108	−0.141	−0.364
Lone-parent family	−3.050	−3.535	1.029	0.970
YP hampered by disability	0.752	−2.614	1.036	−0.624
2^nd^ year in second-level school	1.879	0.358	−0.010	0.673
Neighbourhood disorder at 9	0.455*	0.501*	−0.043	0.131
Family living locally	−1.742	0.636	−0.804	−0.400
SDQ externalising behaviour at 9	−0.151	0.148	−0.001	0.168
SDQ internalising behaviour at 9	−0.688**	−0.499	0.053	−0.019
*Pandemic experiences*				
Quiet place to study	0.546	−0.012	−0.810*	−0.182
Suitable device/computer	0.515	−0.124	0.066	−0.892
Support for learning at home	−1.373	0.469	−0.009	0.194
Fall in household income	−1.297	−2.869*	0.381	−0.548
Received PUP	0.223	1.716	−0.209	−0.008
Parents working remotely:				
One	0.489	−0.598	−0.746	0.228
Both	−1.658	−0.467	0.002	−0.218
Sees friends (Ref. About the same):				
More often	1.690	−1.609	0.282	0.561
Less often	−0.547	−2.344*	−0.238	−0.403
Uses screen for fun more than previously	0.377	−0.185	0.873*	0.799
Engaged in sports:				
More often	1.733	−0.277	0.325	0.368
Less often	0.912	−1.651	−0.152	0.011
Argue with parents more than usual	1.499	−2.268	−0.233	0.086
Argue with siblings more than usual	−0.097	2.490*	0.371	0.564
Worried about family being infected:				
Always true	0.277	1.005	−0.478	−1.075
Sometimes true	1.569	−2.116	−0.982*	−0.875
MHI-5 during pandemic	0.337***	0.443***	−0.132***	−0.177***
Constant	53.366***	44.269***	15.038***	18.616***
Observations	1124	1224	753	851
R-squared	0.222	0.328	0.289	0.306

Notes: OLS regression (robust standard errors)

*** p<.001, ** p<.01, * p<.05; YP=young person

**Fig 2:**
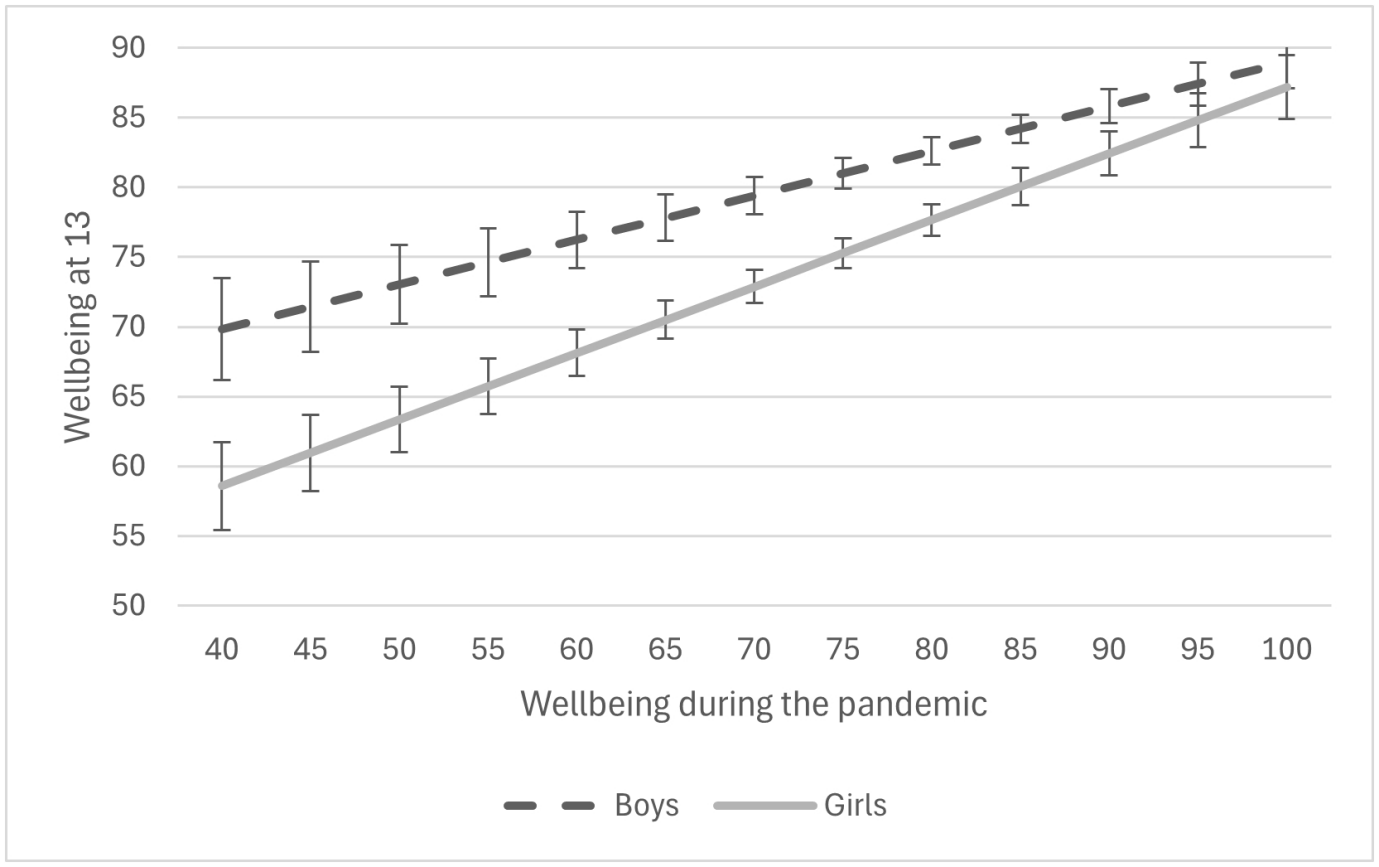
Predicted MHI-5 scores among males and females at age 13 (late-/post-pandemic) by MHI-5 at age 12 (peri-pandemic).

To explore the implications of this difference, Fig 2 plots predicted late-/post-pandemic wellbeing scores (MHI-5) across adolescents’ level of pandemic-period wellbeing (MHI-5) but subdivides this by gender (based on a fully interacted model). Previously, the results demonstrated that girls have lower late-/post-pandemic wellbeing than boys, even after accounting for wellbeing during the pandemic, suggesting the gender gap in wellbeing has widened in the late- to post-pandemic period. Fig 2 demonstrates that this is conditional on young people’s wellbeing during the pandemic. Girls who had high wellbeing during the pandemic exhibit no significant difference in later wellbeing from boys. However, girls who had much lower levels of wellbeing during the pandemic have lower late-/post-pandemic wellbeing than boys who had equally low levels of wellbeing during the pandemic. In other words, in the late-/post-pandemic period, the gender gap in wellbeing has widened but primarily among those who had lower wellbeing during the pandemic.

The second key finding to emerge from the gender analysis is that some pandemic-stressors operate differently for boys and girls (we report gender differences that testing shows are significant at the p < .05 level). For wellbeing (Models 1 and 2, Table 4), girls appear more sensitive to pandemic-stressors than boys, with a pandemic-related fall in household income, seeing friends less often and arguing more with siblings significantly associated with girls’ wellbeing but not boys’ wellbeing. In addition, examining the r-squared scores of the models for boys and girls, the modelled factors explain more of the variance in girls’ wellbeing (33 per cent) than boys’ wellbeing (22 per cent). In contrast to wellbeing, boys’ depression appears more sensitive to pandemic-stressors than girls’ depression. Not having a quiet place to study and increased screen time are both associated with higher depressive symptoms among boys but not girls. The factors modelled here also explain a similar amount of the variance in boys’ and girls’ depressive symptoms (29 per cent and 31 per cent respectively). Table 4 also reveals how boys and girls from different types of homes/communities may have experienced the pandemic differently, potentially shaping their mental wellbeing differently. For example, girls who passed the pandemic in more urban areas report worse late-/post-pandemic mental wellbeing than boys in urban areas. Mothers’ education also appears more strongly linked to girls’ mental wellbeing than boys. The following section will discuss the key findings and implications of the study.

## Discussion

External shocks, such as global recessions, have been found to lead to scarring effects on young people’s wellbeing (see, for example, [38]). The pandemic and related public health restrictions can be regarded as a massive and protracted external shock. A good deal of research has focused on adult and youth wellbeing during the pandemic, especially the first period of closures, but there is relatively little information on whether (and which) stressors experienced at the height of the pandemic have shaped longer-term wellbeing. This study contributes to the existing body of research by looking at adolescent wellbeing in the late/post-pandemic period. While young people shared some common experiences during the pandemic, the study exploits important variation in the impact on the family economic situation, familial and peer interaction, and engagement in remote learning to examine whether these pandemic stressors have any longer-term associations with adolescent mental wellbeing outcomes in Ireland, as the country emerged from a period of protracted restrictions.

Previous research [12,13], as well as analyses conducted here, found that, during the pandemic, adolescents reported lower wellbeing during the pandemic in response to a range of pandemic-stressors. This study reveals several key findings regarding how these stressors are linked with mental wellbeing in the longer-term.

The first key finding is that several stressors do have a long-term association with young people’s mental wellbeing. Economic pandemic-stressors appear important, with adolescents in households that, during the pandemic, reported a decline in income or who did not receive PUP payments still exhibiting lower late-/post-pandemic wellbeing (MHI-5 but not SMFQ depressive symptoms). This may be because these families’ economic situations have still not recovered (although the relationship remains even after accounting for late-/post-pandemic household income or perceived financial security). It may also be that the pandemic-experiences of pecuniary hardship caused significant parental stress, which can have a lasting association with young people’s wellbeing, even after the economic situation has recovered (of course, the cost-of-living crisis started during the second half of the fieldwork period; however, additional analyses suggest accounting for post-pandemic financial strain does not change our findings) [38]. Not having a quiet place to study during the pandemic is also associated with long-term depressive symptoms. This could be driven by its relationship with educational development with young people falling further behind during the pandemic, exhibiting a persistent link with their mental wellbeing [35]. Alternatively, it could be driven by housing conditions, such as overcrowding, which appear linked with longer-term indicators of poorer mental wellbeing [50].

The results also show that the pandemic’s disruption to young people’s social relations has long-term implications for mental wellbeing. Young people who saw their friends less than usual (even after having returned to school in December 2020) report lower late-/post-pandemic wellbeing. One possibility is that their late-/post-pandemic social networks had still not recovered. It may also demonstrate that disruption during a key developmental stage, where young people develop social skills with peers and are exposed to new peer groups with the transition to secondary school, is continuing to hamper their peer relations late-/post-pandemic, resulting in persistently lower mental wellbeing [6]. Adolescents who argued with their parents more during the pandemic also report worse late-/post-pandemic wellbeing, suggesting that more time in conflictual familial environments, or experiencing harsher family dynamics, may leave a lasting scar on youth mental wellbeing [34]. In addition, young people who increased their screen time during the pandemic report more late-/post-pandemic depressive symptoms, which may have led to longer-term internalising symptoms [37] or elevated screen time use late-/post-pandemic [36]. Ultimately, these findings support the ‘vulnerability–stress’ model which suggests repeated experiences of stress can have compounding, long-term negative impacts on mental wellbeing [16].

The second key finding is that some pandemic-stressors appear linked with long-term mental wellbeing via their association with young people’s mental wellbeing during the pandemic itself. The associations between late-/post-pandemic mental wellbeing outcomes and arguing with parents, lower connectivity with friends or not having a quiet place to study are all reduced after accounting for wellbeing during the pandemic. This suggests part of the apparent long-term associations between the pandemic and mental wellbeing may come through the association between prior mental wellbeing and future mental wellbeing, such as persistent changes in beliefs about oneself and the world [35]. However, some pandemic-stressors, such as household income shocks or increased screen time, appear independently associated with young people’s late-/post-pandemic mental wellbeing outcomes, even after accounting for peri-pandemic wellbeing. The third key finding is that some pandemic-stressors that were linked with mental wellbeing during the pandemic are not significantly associated with late-/post-pandemic mental wellbeing outcomes. Education-related stressors are no longer significantly associated with late-/post-pandemic mental wellbeing, potentially suggesting that once children returned to school, the wellbeing of those who lacked educational resources during the pandemic recovered. In addition, anxiety around family COVID-19 infection no longer predicts mental wellbeing in the late-/post-pandemic period, suggesting that once the fear of infection lessened such anxiety matters less for mental wellbeing.

The study also shows important gender differences in late-/post-pandemic mental wellbeing. As discussed, prior research demonstrates that girls experienced worse pandemic mental wellbeing outcomes than boys [5,7,20,23]. This study finds that this gender gap has widened into the late-/post-pandemic period. However, it also finds this gap is particularly pronounced among girls whose mental wellbeing was especially poor during the pandemic. Girls whose wellbeing remained much higher during the pandemic see little or no difference to boys in the late-/post-pandemic period. Boys and girls were sensitive to different stressors. Of particular note is the stronger long-term negative association between a lack of seeing friends during the pandemic and wellbeing among girls, even after accounting for peri-pandemic wellbeing. Potentially, girls tend to rely on peers more for emotional support when dealing with life stressors and being disconnected from peers may have removed a key support mechanism [25].

Overall, some stressors experienced during the pandemic appear to have left a lasting mark on both dimensions of young people’s mental wellbeing: wellbeing and depressive symptoms. However, there are differences in how different stressors are related to each dimension. One possibility is that the wellbeing measure captures both positive and negative affect while the depression scale focuses on negative emotions only. While significantly related, the correlation is only moderate in strength, and the two measures may be capturing different aspects of overall wellbeing.

Despite its insights, the study has limitations. The first issue is that the data did not contain identical pre-, peri- and late-/post-pandemic measures of mental wellbeing. Although not the primary aim of this study, this limitation meant we could not track changes in mental wellbeing over time. This is because measures designed to tap mental wellbeing among twelve and thirteen-year-olds (MHI-5; SMFQ) are not designed to be asked of nine-year-olds, for which we had to use measures of internalising/externalising socioemotional problems (although a key advantage to using these measures is that they are self-reported by adolescents and not reliant on parental views of their children’s wellbeing). Therefore, the models do not control for pre-pandemic mental wellbeing using identical measures to late-/post-pandemic mental wellbeing. As such, pre-pandemic mental wellbeing could still affect the extent to which children reported certain pandemic-period stressors. In addition, the pandemic-period survey only included the MHI-5 measure of wellbeing. Therefore, models of late-/post-pandemic SMFQ could not account for pandemic-period depressive symptoms. Furthermore, MHI-5 was collected online during the pandemic but by phone in the late-/post-pandemic period. Given respondents may be more inclined to respond positively via phone interviews, estimates of late-/post-pandemic MHI-5 may be positively biased. These limitations also mean more robust, longitudinal fixed-effects approaches could not be undertaken, accounting for time invariant unobserved heterogeneity, which could therefore bias estimates while the risk of bias remains from time-variant unobserved heterogeneity. For example, we cannot fully rule out the possibility that the observed associations reflect unobserved stability in adolescents’ mental wellbeing or family environments rather than apparent scarring effects of pandemic stressors. In particular, unmeasured characteristics, such as personality traits, family dynamics, or coping capacities, may influence both exposure to certain pandemic experiences and later wellbeing outcomes. In addition, the current methodological approach does not completely eliminate the possibility of reverse causality, whereby adolescents with poorer wellbeing during the pandemic may have been more likely to experience or report certain stressors. As such, the findings should be interpreted as identifying associations between pandemic experiences and later wellbeing rather than definitive causal effects.

Child-to-adolescent transitions are also linked with developmental changes in mental wellbeing. Although the current study was not tracking absolute changes in mental wellbeing, it is important to keep in mind that certain stressors may have had particular relationships with mental health given underlying developmental trends in youth wellbeing. Bias may also be introduced from attrition over time in the data. While the longitudinal weights applied go some way towards addressing this, where attrition may be linked to particularly negative impacts of the pandemic, such groups may be absent from the data, potentially underestimating the more acute relationships between the pandemic and wellbeing. Lastly, the finding of a widening gender gap in mental wellbeing outcomes in the late-/post-pandemic period cannot account for potential differences in gender-specific trends in mental wellbeing that occur as children grow up.

Any estimates of the long-term associations between the pandemic and mental wellbeing must also consider how the late-/post-pandemic period also saw several other crises (e.g., inflation, rising costs of living, the war in Ukraine, etc.) which could feasibly be shaping young people’s late-/post-pandemic mental wellbeing. While some robustness tests (e.g., accounting for perceived financial situation in the late-/post-pandemic period) did not account for the observed role of pandemic-stressors, the possibility remains that the emergence of subsequent, late-/post-pandemic crises may be exacerbating the longer-term relationships between the pandemic and wellbeing, for example, by inhibiting processes of recovery.

In sum, this study suggests the COVID-19 pandemic may have had long-term negative impacts on adolescent mental wellbeing, particularly via economic stressors and disruption to peer and familial relations. Part of the long-term association comes through how pandemic-stressors are linked with mental wellbeing during the pandemic itself, which continue to shape young people’s long-term mental wellbeing trajectories. However, some pandemic-stressors, such as family income shocks during the pandemic, evidence persistent longer-term associations with young people’s mental wellbeing. These findings have important implications for public health and youth policy. If a substantial cohort of young people experienced persistently worse wellbeing following the pandemic, particularly girls and those in economically vulnerable households, this raises concerns about the longer-term developmental consequences of large societal shocks. Adolescence is a critical developmental period during which mental wellbeing can shape educational trajectories, social development and later-life health. The persistence of poorer wellbeing among some groups therefore suggests the need for sustained recovery strategies rather than assuming that young people will automatically return to pre-pandemic trajectories. Indeed, there is evidence in Ireland of a significant rise in school absence post-pandemic, especially in schools serving more disadvantaged communities [51]. Interventions may be needed at multiple levels. At the family level, policies that buffer households from economic shocks, such as income supports or housing stability measures, and that provide holistic services for parents with mental health difficulties may indirectly protect young people’s mental wellbeing. At the school level, targeted wellbeing and mental health supports, peer-connection programmes and resources for students experiencing social or academic disruption may help mitigate longer-term impacts. More broadly, these findings underline the importance of incorporating youth wellbeing into preparedness planning for future societal crises, ensuring that social, educational and economic policies are designed with adolescents’ developmental needs in mind.

## Supporting information

S1 Appendix Factors associated with wellbeing (MHI-5) during the pandemic (at age 12), controlling for internalising and externalising behaviour at age 9. (DOCX)
