# Supplementary material for: COVID-19 pandemic stressors and their longer-term association with young people’s wellbeing
Source: PLoS One. 2026 May 13;21(5):e0347875. doi: 10.1371/journal.pone.0347875 (PMC13170836; doi:10.1371/journal.pone.0347875)
Supplement: S1 Appendix — (DOCX) [file pone.0347875.s001.docx]

**Appendix S.1 - Factors associated with wellbeing (MHI-5) during the pandemic (at age 12), controlling for internalising and externalising behaviour at age 9**

|  | Model 1 |
| --- | --- |
| *Pandemic Outcome:* | MHI-5 |
|  |  |
| Female | -5.145*** |
| Mother’s education |  |
| Leaving Certificate | -0.560 |
| Post-secondary | -1.849 |
| Degree or higher | -1.889 |
| Income at age 9: |  |
| Quintile 2 | -0.856 |
| Quintile 3 | -0.581 |
| Quintile 4 | -0.346 |
| Quintile 5 | -0.363 |
| Income missing | 1.041 |
| Urban | -2.419** |
| Migrant background | -1.265 |
| Lone-parent family | -1.468 |
| YP hampered by disability | -1.712 |
| 2^nd^ year in second-level school | -1.896** |
| Neighbourhood disorder at 9 | 0.280 |
| Family living locally | 1.583* |
| SDQ externalising behaviour at 9 | -0.066 |
| SDQ internalising behaviour at 9 | -0.680*** |
| *Pandemic experiences* |  |
| Quiet place to study | 3.619*** |
| Suitable device/computer | 3.678*** |
| Support for learning at home | 2.411** |
| Fall in household income | -3.201*** |
| Received PUP | 2.671** |
| Parents working remotely: |  |
| One | -2.565** |
| Both | -1.805 |
| Sees friends (Ref. About the same): |  |
| More often | 0.359 |
| Less often | -1.917* |
| Uses screen for fun more than previously | -3.311*** |
| Engaged in sports: |  |
| More often | -0.947 |
| Less often | -0.801 |
| Argue with parents more than usual | -7.378*** |
| Argue with siblings more than usual | -0.730 |
| Worried about family being infected: |  |
| Always true | -6.010*** |
| Sometimes true | -2.488* |
|  |  |
| Constant | 85.977*** |
| Observations | 2424 |
| R-squared | 0.265 |

Notes: YP=young person; OLS regression (robust standard errors)

*** p<.001, ** p<.01, * p<.05
